# Pre-exposure Prophylaxis (PrEP) Information on Instagram: Content Analysis

**DOI:** 10.2196/23876

**Published:** 2021-07-27

**Authors:** Eric Walsh-Buhi, Rebecca Fagen Houghton, Claire Lange, Ryli Hockensmith, John Ferrand, Lourdes Martinez

**Affiliations:** 1 Department of Applied Health Science Indiana University School of Public Health-Bloomington Bloomington, IN United States; 2 School of Communication San Diego State University San Diego, CA United States

**Keywords:** digital health, social media, HIV, pre-exposure prophylaxis, Instagram, content analysis, communication

## Abstract

**Background:**

There is still an HIV epidemic in the United States, which is a substantial issue for populations bearing a disproportionate burden of HIV infections. Daily oral pre-exposure prophylaxis (PrEP) has proven to be safe and effective in reducing HIV acquisition risk. However, studies document that PrEP awareness/usage is low. There is also limited understanding of social media platforms, such as Instagram, as PrEP information sources.

**Objective:**

Given the paucity of research on PrEP-related Instagram posts and popularity of this social media platform, the purpose of this research is to describe the source characteristics, image types, and textual contents of PrEP-related posts on Instagram.

**Methods:**

Using Crowdtangle Search, a public insights tool owned/operated by Facebook, we retrieved publicly accessible and English-language-only Instagram posts for the 12-month period preceding April 22, 2020, using the following terms: Truvada or “pre-exposure prophylaxis” or #truvada or #truvadaprep or #truvadawhore or #truvadaforprep. We employed a qualitative coding methodology to manually extract information from posts. Using a pretested codebook, we performed content analysis on 250 posts, examining message and source characteristics (ie, organization type [eg, government, news] and individual type [eg, physician]), including information about PrEP (eg, how it works, cost), and indicated users. Frequencies and percentages were calculated for all categorical variables. A Chi-square test was conducted to determine differences between source types on a variety of message characteristics.

**Results:**

Three-quarters of the posts (193/250, 77.2%) were posted by organizations. Of the 250 posts reviewed, approximately two-thirds (174/250, 69.6%) included a photograph, more than half (142/250, 56.8%) included an infographic, and approximately one-tenth (30/250, 12%) included a video. More than half defined PrEP (137/250, 54.8%), but fewer posts promoted PrEP use, explained how PrEP works, and included information on the effectiveness of PrEP or who can use it. The most commonly hashtagged populations among posts were men who have sex with men (MSM), but not necessarily bisexual men. Few posts contained race-/ethnicity-related hashtags (11/250, 4.4%). Fewer posts contained transgender-associated tags (eg, #transgirl; 5/250, 2%). No posts contained tags related to heterosexuals or injection drug users. We found statistical differences between source types (ie, individual versus organization). Specifically, posts from organizations more frequently contained information about who can use PrEP, whereas posts from individuals more frequently contained information describing adverse effects.

**Conclusions:**

This study is among the first to review Instagram for PrEP-related content, and it answers the National AIDS Strategy’s call for a clearer articulation of the science surrounding HIV risk/prevention through better understanding of the current public information environment. This study offers a snapshot of how PrEP is being discussed (and by whom) on one of the most popular social media platforms and provides a foundation for developing and implementing PrEP promotion interventions on Instagram.

## Introduction

### Background

There is still an HIV epidemic in the United States, which is a substantial issue for priority populations bearing a disproportionate burden of HIV infections. In 2018, rates of HIV diagnoses remained higher among gay and bisexual men (GBM) and other men who have sex with men (MSM), with a particularly high burden among Black GBM; Black and Latinx women and men; people who inject drugs; people aged 20-34 years; people in the southern parts of the United States; and transgender women, with a high burden among Black transwomen [1,2].

### PrEP for HIV Prevention

Daily oral pre-exposure prophylaxis (PrEP), with a fixed-dose combination of tenofovir disoproxil fumarate (TDF) 300 mg and emtricitabine (FTC) 200 mg, has proven safe and effective in reducing the risk of sexual HIV acquisition in adults [3]. Several PrEP clinical trials have demonstrated its safety and a substantial reduction in the rate of HIV acquisition in MSM, men and women among heterosexual HIV-discordant couples, heterosexual men and women recruited as individuals, and persons who inject drugs [4-7]. Accordingly, PrEP is recommended for three main adult groups in the United States—MSM, heterosexually active adults, and persons who inject drugs—estimated in 2015 at 1.1 million [8].

### PrEP Awareness and Usage

Large national studies of GBM, transmasculine individuals, and Black people in the United States document that PrEP awareness and usage are low [9-11]. For example, in a probability-based cohort study of gay and bisexual men, Dodge et al reported that among HIV-negative/unknown/untested GBM, <7% reported using PrEP in the past 6 months [12]. Over half of the GBM reported not using a condom during anal sex with their most recent partner, with 94% of these men not on PrEP. Among high-risk Black individuals participating in another study, one-fifth knew about PrEP and the most reported reason for the lack of willingness to use PrEP was low self-perceived risk (65%). In urban areas, although PrEP awareness and use among MSM has increased, PrEP use remains lower among Black and Latinx MSM [13]. Lastly, PrEP awareness and use is critically low among persons who inject drugs [14-17].

In 2015, the National HIV/AIDS Strategy for the United States updated to 2020 was released by the White House and included four key areas [18]. One critical area is “full access to comprehensive PrEP services for those whom it is appropriate and desired, with support for medication adherence for those using PrEP.” The strategy outlined the need for providing “clear, specific, consistent, and scientifically up-to-date messages about HIV risks and prevention strategies” and utilization of “evidence-based social marketing and education campaigns, and…(leveraging of) digital tools and new technologies,” such as social media platforms [18].

### PrEP and Social Media

Although PrEP is a novel and promising approach to HIV prevention, there is limited understanding of social media platforms as sources of information on PrEP. Social media platforms are interactive websites and applications that enable users to create, share, comment on, and modify content [19]. As of 2019, 9 in 10 US adults reported that they use the Internet, 8 in 10 reported that they own a smartphone, and 7 in 10 reported that they use social media [20]. Black and Latinx individuals; lesbians, gay men, and bisexuals; and young adults who are at the greatest risk for HIV are heavy users of social media, and they represent important priority populations for HIV risk/prevention messages [21,22].

Twitter and, to a lesser extent, YouTube appear to be the preferred social media platforms for most research on PrEP. For example, Kecojevic et al examined firsthand narratives of individuals detailing their PrEP experiences via YouTube [23,24]. They reported that the narratives covered a wide range of topics (eg, reasons to start PrEP, interacting with providers, PrEP adverse effects, insurance coverage, stigma); although the study focused on gay men, the authors emphasized a general lack of PrEP awareness in the lesbian, gay, bisexual, and transgender community. Using natural language processing techniques, Breen et al found increasing PrEP discussion on Twitter [25]. They further noted that people who mention PrEP on Twitter also talk about other issues, including general health, sexually transmitted diseases (STDs), stigma, and politics. McLaughlin et al performed a content analysis of PrEP for HIV prevention on Twitter and found that the most common categories of tweets pertained to recipients of PrEP, with the second and third most common categories capturing efficacy and moral judgment, respectively [26]. The authors also found that individuals (as opposed to organizational accounts) comprised the majority of tweet creators. In their content analysis of PrEP messaging on Twitter, Schwarz and Grimm found that 54% of the tweets included PrEP awareness/information, 15% discussed the barriers to use PrEP, 14% contained consequences/limitations, 9% included antistigma, and 6% mentioned the stigma of using PrEP [27]. They further reported that tweets mentioning race, gender, and sexual orientation were rare. Individuals were more likely to tweet about antistigma than organizations and media outlets.

Although research has examined PrEP messaging on Twitter, to date, we have found limited PrEP-related research focusing on popular image-sharing social media sites such as Instagram. With more than 1 billion monthly active users, Instagram is an increasingly popular photo- and video-based social media platform with a wide variety of users [28]. Each day, Instagram users upload more than 500 million photos, videos, and stories (a combination of videos, text, and photos) [29]. Instagram is the third most popular social media platform in the United States, behind YouTube and Facebook, with 38% and 67% of US adults and 18-29-year-olds, respectively, using Instagram [21]. It is also an excellent platform for reaching out to a diverse population, as the platform has a balance of genders (52% female), and 40% of Black and 51% of Latinx Internet-using adults use Instagram [21,29]. This positions Instagram as a unique platform for examining health communication and, specifically, PrEP-related communication intended for and among racial and ethnic minority priority populations.

Instagram previously has been examined as a health promotion modality with some researchers highlighting the general utility of Instagram as a source of education and motivation [30] as well as users’ experiences on receiving social support via Instagram [31]. Additionally, Instagram relies heavily on images in its posts and has been referred to as a forum for parasocial interaction or the one-sided feeling of connectedness between a fan and a celebrity [32]. Parasocial interaction can be examined in the context of Instagram posts owing to the large fan bases (ie, followers) attached to certain Instagram celebrities as well as those celebrities’ depictions of their personal lives through images. Given that other social media sites are not exclusively image-driven, this positions Instagram as an optimal data source for this type of study.

In one study, Nobles et al examined Instagram posts from January 2017 to July 2018; although they did not focus specifically on PrEP, they did find that PrEP was mentioned in a very small percentage (6.2%) of the posts tagged “HIV” [33]. These authors further noted that the visual content of specific clinical interventions, such as PrEP promotion, are not well represented on Instagram relative to public health priorities. To date, only one study [34] has been undertaken to specifically examine Instagram posts in the context of PrEP promotion efforts, and its results describe surface-level engagements in Instagram posts as generally positive among Black MSM. Given the paucity of research on PrEP-related Instagram posts and the popularity of this social media platform, the purpose of this research is to build on previous research, such as the study by Nobles et al (which is broad in focus and characterizes the social media landscape regarding HIV risk and prevention messaging), with particular focus on PrEP-related communications on Instagram. Although past research suggests that PrEP awareness/information comprises the largest content focus on Twitter [27] and that most PrEP-related content on YouTube focuses on GBM or MSM [23,24,35], it remains unclear whether these result patterns exist on Instagram as well. Based on this, we pursued three research questions (RQs):

*RQ1: What is the textual content of PrEP-related Instagram posts?*

*RQ2: Which priority populations are the focus of PrEP-related Instagram posts?*

*RQ3: How does the textual content of PrEP-related posts vary by source (ie, individual accounts versus organizational accounts)?*

Attempting to answer these research questions will allow us to describe the landscape surrounding PrEP-related posts on a social media platform that has been understudied in the context of sexual health research and how posts are designed to target specific users. These answers will be used to develop novel communication strategies for promoting PrEP uptake and adherence in populations at risk for HIV transmission.

## Methods

### Data Source

Using Crowdtangle Search [36], a Facebook-owned tool that tracks interactions on public content from Facebook pages and groups, verified profiles, Instagram accounts, and subreddits, we retrieved publicly accessible and English-language-only Instagram posts for the 12-month period preceding April 22, 2020, using the following search terms: Truvada or “pre-exposure prophylaxis” or #truvada or #truvadaprep or #truvadawhore or #truvadaforprep [36]. We selected these hashtags based on previous Twitter data research and a review of popular PrEP-related hashtags provided in the Instagram search textbox as well as those that were suggested by the search textbox when our initial terms were entered. The initial search returned 275 posts. A total of 250 posts were analyzed, as 20 posts were excluded for not being in English and 5 for nonfunctioning links at the time of coding. Data on the final 250 public posts included the post URL, account and username associated with the post, date and time of posting, type of post (eg, photo, video), number of followers at the time of posting, and text associated with the post. Engagement metrics were also available and analyzed, including the number of likes as well as the number of comments and views.

### Data Coding

Despite the growing use of machine learning methods, there is evidence that these methods do not always align well with social science objectives [37]. For this study, we opted to use a qualitative coding methodology through the development of a coding document adapted from sentiment and other content analysis research (eg, PrEP on YouTube, human papillomavirus [HPV] vaccine information on YouTube) to manually extract information from this sample of Instagram posts [23,24,38-40]. Using a pretested codebook, we performed content analysis on all the 250 posts. The codebook included variables related to the sources, image types, and caption characteristics, as well as hashtags used for each post. The coded source characteristics included whether the Instagram account/profile represented an individual or organization. Individual profiles were coded according to self-identification as a parent, child, or spiritual/religious person; political affiliations; and their reported professions (eg, journalist, physician). For organizational accounts, the organization type was coded using information displayed in the user profile or links embedded in the user profile or bio to the organization’s website (eg, business, media outlet, nonprofit, government). As we extracted data from the biographies/profiles of the posters, these data are not considered anonymous. For example, some “personal” information was recorded, such as the name (eg, Prevention305) and affiliation of the poster.

Guided by previous research, we coded the Instagram posts for specific information about PrEP and the indicated users [23,24] as follows: whether the posts defined PrEP, explained how PrEP works, who can use it, or how to obtain it; discussed the effectiveness of using PrEP to prevent HIV; mentioned the adverse effects of using PrEP; promoted PrEP use; and indicated any stigmatization or antistigma sentiments regarding PrEP users (eg, “My pharmacist just called me a slut for taking Truvada.”) [23,27]. The definitions for the variables mentioned above are listed in Table 1.

**Table 1:** Definitions, examples, and frequencies of textual content characteristics and variables regarding pre-exposure prophylaxis–related messages (N=250).

Message characteristic	Definition	Example Instagram post	n	%
Defines PrEP^a^	Taking a prescription drug as a means of preventing HIV^b^ infection in an HIV-negative person	“Truvada and Descovy are FDA^c^-approved medications to prevent HIV when taken once daily.”	137	54.8
Promotes PrEP use	Encouraging PrEP use among those at risk, can include action items, such as “ask your doctor about PrEP.”	“Talk to your doctor or local clinician to determine if PrEP might be right for you.” “#PrEP2BeSafe like the #MenOfPrEP with your NEW HAPPY PILL”	93	37.2
How PrEP works	By taking Truvada/PrEP (a combination of two drugs, tenofovir and emtricitabine) daily, the presence of the medicine in the bloodstream can stop HIV from taking hold and spreading in the body.	“PrEP involves taking the combination drug emtricitabine-tenofovir (Truvada) or emtricitabine plus tenofovir alafenamide (Descovy) every day.” “Having PrEP medicine in your bloodstream can stop HIV from taking hold and spreading in your body.”	80	32.0
Effective in preventing HIV	Discusses the effectiveness of PrEP, such as successful prevention of HIV and statistics on prevention	“When taken daily, PrEP pills can reduce the risk of contracting HIV through sex by about 99 percent.”	70	28.0
Who can use PrEP	Claiming or mentioning who should receive PrEP (ie, MSM^d^, “high-risk” individuals)	“Pre-exposure prophylaxis (or PrEP) is a daily medication that allows people at very high risk for HIV to lower their chances of getting infected.” “PrEP has been shown to reduce risk of HIV infection through sex for gay and bisexual men, transgender women, and heterosexual men and women, as well as among people who inject drugs.”	68	27.2
How to obtain PrEP	Mentioning where or how to obtain PrEP	“The Ready, Set, PrEP program makes PrEP medication available at no cost for qualifying recipients. To receive PrEP medication through this program, you must:⠀ -Lack prescription drug coverage⠀ -Be tested for HIV with a negative result⠀ -Have a prescription for PrEP⠀ Talk to your health care provider or find a provider at HIV.gov Locator to find out if PrEP is right for you.”	55	22.0
Costs of PrEP	Mentioning the cost of PrEP or if insurance covers it	“The Ready, Set, PrEP program makes PrEP medication available at no cost for qualifying recipients.”	49	19.6
Antistigma	Owning the words, re-appropriate use, critique the use of stigma (eg, Truvada whore)	“The provision of the pill not only helps to reduce infections but allows for vulnerable populations and those often under a stigma, the opportunity to have access and be even more safe.”	20	8.0
Adverse effects	Mentioning the adverse effects of consuming PrEP/Truvada (ie, kidney damage)	“Have you or a loved one taken the antiretroviral, Truvada, and experienced osteoporosis, kidney failure, and broken or brittle bones?”	17	6.8
Stigmatization of PrEP users	Stigmatization of PrEP users, and negative attitudes or beliefs directed toward PrEP users	“Azealia Banks apologizes for ‘extremely insensitive’ comments on PrEP meds – ‘gay men should just be responsible, so they don’t have to take a f*cking pill’”	5	2.0

^a^PrEP: pre-exposure prophylaxis.

^b^HIV: human immune virus.

^c^FDA: Food and Drug Administration.

^d^MSM: men who have sex with men.

Based on previous research, we also coded for whether each post included any race-associated hashtags (eg, #blacklove), male-associated tags (eg, man, boy, male), female-associated tags (eg, woman, girl, female), transgender-associated tags (eg, #trans, transgirl), and any other tags related to the PrEP guidelines of the Centers for Disease Control and Prevention (CDC) (eg, #lgbt, #lgbtq, #gay, #queer, #heterosexual, #drugs) [3,41]. Previous research highlights the utility of hashtags in reaching a wider audience, so we determined that this would be an effective way to identify the target audiences of these posts [42,43]. The raters relied on hashtags and captions to identify the target audience and did not attempt to code the physical characteristics of those featured in photographs, as assuming one’s ethnic background or gender identity did not seem appropriate. Finally, we coded for the source that the post credited (eg, CDC, medical doctor), and whether the posts gave firsthand accounts of experiences with PrEP, whether the person writing the post was a child or an adult, and whether the firsthand experience with PrEP originated from an individual who belongs to the MSM group. We identified over 500 unique hashtags. Table 2 displays the top 10 most frequently used hashtags in this sample.

**Table 2:** Ten most common hashtags found in pre-exposure prophylaxis–related Instagram posts (N=541).

Hashtag	Count, n	%
#prep	41	7.6
#hiv	39	7.2
#hivprevention	32	5.9
#truvada	22	4.1
#gayman	16	3.0
#gaytheringhotel	13	2.4
#descovyprep	12	2.2
#truvadaprep	12	2.2
#sexualhealth	11	2.0
#aids	9	1.7

Two raters independently coded 57 characteristics of the posts in multiple stages. Specifically, the raters coded a set of Instagram posts after which the raters and a moderator discussed and resolved the discrepancies between ratings. This process was repeated for a total of four coding stages wherein each post was double coded on each variable. This was done to identify issues with the coding protocol and other errors. For instance, 1 post from the first set of 20 posts ultimately resulted in a broken link on the date which the raters met to reconcile their codes and was ultimately excluded from the final data set. After the first discussion on the coding differences and questions about the interpretation of the codebook definitions, the codebook was revisited with feedback from the raters to make the definitions more specific. Next, the two raters independently coded another 42 Instagram posts and repeated the process. Then, the raters independently coded another 40 Instagram posts and met to discuss coding differences. This process was then repeated with the remaining 149 Instagram posts. The codes from these four iterative rating stages were merged to form a data set of 250 posts. The lowest observed Cohen kappa (κ) statistic found in this merged data set was -0.02, which occurred when the raters attempted to code for Stigma of PrEP Users present in the posts. Only 2 variables reported κ values that were less than 0.21, which indicates fair or higher levels of agreement across the various observations [44]. The median κ value was 0.76. After identifying the discrepant codes, the two raters and the moderator met to reconcile the codes for each variable in this data set and reached a perfect agreement over the final data set.

### Data Analysis

We conducted all analyses using IBM SPSS Statistics (Release 26.0.0.0) and R (Foundation for Statistical Computing, Vienna, Austria; https://www.R-project.org/). The measures of central tendency (eg, mean, median) and dispersion (eg, standard deviation [SD]) were calculated for the continuous variables. Frequencies and percentages were calculated for all the categorical variables. Lastly, a Chi-square test was conducted to determine the statistical differences between source types (ie, individual versus organization) regarding a variety of other characteristics, including those displayed in Tables 1 and 3.

**Table 3:** Source attribution in pre-exposure prophylaxis–related Instagram posts (N=250).

Source attribution: Posts citing information from the sources listed below	n	%
The Centers for Disease Control and Prevention or other another federal entity or foreign equivalent	25	10.0
PrEP^a^ manufacturer (eg, Gilead, Merck)	22	8.8
Government officials (eg, senators, governors, representatives) or from political organizations	17	6.8
A celebrity	13	5.2
Another source such as WebMD, Mayo Clinic	11	4.4
A state or local health department	9	3.6
A medical doctor (physician)	8	3.2
A member of the research community (eg, researcher, scientist)	7	2.8
The World Health Organization	2	0.8

^a^PrEP: pre-exposure prophylaxis.

## Results

### Descriptive Statistics

For the 250 reviewed PrEP-related Instagram posts, the mean number of likes was 844.5 (SD 3,596.2; median 43.5; range 1-45,674); the mean number of comments was 16.96 (SD 77.1; median 1; range 0-1,086). For the 30 video posts, the mean number of views was 1033.4 (SD 6,523.3; median 0; range 0-64,866). Data for the number of followers were available for 193 posts; the mean number of followers was 114,487.6 at the time of posting (SD 598,905.8; median 11,705; range 459-7,780,734). As we focused on analyzing the textual content of the Instagram posts, we found that the text character counts varied. The mean number of characters in the posts was 645.4 (SD 467.1; median 515; range 1-2,196).

### Source Characteristics

More than 75% of all the posts (193/250, 77.2%) were posted by organizations, whereas 22% (55/250) were posted by individuals. The remaining posts came from Instagram accounts that were unclear in terms of whether they were representing an individual or organization. Among the 193 Instagram posts made by organizations, the largest identified types were health information providers (115/193, 59.6%), nonprofit/advocacy groups (95/193, 49.2%), and health care organizations (89/193, 46.1%). Among the 55 posts made by individuals, the largest identified types were those having a political affiliation (6/55, 10.9%), journalists (5/55, 9.1%), nurses or allied health workers (5/55, 9.1%), and physicians (4/55, 7.3%), as shown in Tables 4 and 5.

**Table 4:** Source characteristics of Instagram posts among individuals regarding pre-exposure prophylaxis (N=55).

Information available in the posts, biographies, or profiles of individuals	n	%
Politics (eg, political affiliation)	6	10.9
Being a journalist or member of the press	5	9.1
Being a nurse or allied health worker	5	9.1
Being a physician (eg, medical doctor, MD^a^, DO^b^, resident)	4	7.3
Being a health educator	3	5.5
Religion or spirituality (eg, scripture, prayer, god)	2	3.6
Being a child (eg, son or daughter)	1	1.8
Being a parent (eg, mother or father)	0	0
Being an epidemiologist	0	0
A personal account from individuals with a firsthand experience with PrEP^c^, Truvada, or HIV^d^/AIDS^e^	10	18.2
A personal account originating from an individual who is a man who has sex with men (eg, GBM^f^)	15	27.3

^a^MD: Doctor of Medicine.

^b^DO: Doctor of Osteopathic Medicine.

^c^PrEP: pre-exposure prophylaxis.

^d^HIV: human immunodeficiency virus.

^e^AIDS: acquired immunodeficiency syndrome.

^e^GBM: gay and bisexual men.

**Table 5:** Source characteristics of Instagram posts among organizations regarding pre-exposure prophylaxis (N=193).

Information available in the posts, profiles, biographies, or affiliated websites of organizations	n	%
Health information provider	115	59.6
Nonprofit/advocacy group	95	49.2
Health care organization	89	46.1
Business (eg, company, franchise, store, product, or service)	44	22.8
Nonhealth-related advocacy group	42	21.8
News or media organization	40	20.7
City, state, or federal government	16	8.3
School or school district	1	0.5

It must be noted that the source characteristics in Table 4 do not add up to 100% owing to information being unavailable on the individual poster’s Instagram bio. The source characteristics in Table 5 exceed 100% because an organization may be included under multiple categories (eg, a business and a health care organization).

### Media Type

Of the 250 Instagram posts reviewed, more than two-thirds (174/250, 69.6%) included images (ie, nonmoving photo image or snapshot), more than half (142/250, 56.8%) included infographics (ie, photos or images containing factual information/data/charts), and approximately one-tenth (30/250, 12%) included a video (eg, video, Boomerang, graphic interchange format [GIF] files).

### RQs

*RQ1: What is the textual content of PrEP-related Instagram posts?* Table 1 displays the results answering our first research question. We observed that more than half of all the reviewed Instagram posts defined PrEP (137/250, 54.8%). Fewer posts promoted PrEP use (93/250, 37.2%), explained how PrEP works (80/250, 32%), and included information on PrEP’s effectiveness (70/250, 28%) or who can use PrEP (68/250, 27.2%). Less than one-quarter of all the reviewed posts provided information regarding how to obtain PrEP (55/250, 22%), costs related to PrEP (49/250, 19.6%), or adverse effects of PrEP (17/250, 6.8%). More posts were classified as battling stigma (20/250, 8%) than those classified as stigmatizing PrEP users (5/250, 2%). As displayed in Table 3, less than half of all the posts provided some source attribution (eg, citation) for the information posted. The most cited sources in the reviewed posts were the CDC (25/250, 10%) and PrEP manufacturers (eg, Gilead; 22/250, 8.8%).

*RQ2: Which priority populations are the focus of PrEP-related Instagram posts?* When answering our second research question, we found that PrEP-related Instagram posts were most commonly focused on GBM and MSM. The most commonly hashtagged priority population among posts included MSM but not necessarily bisexual men (eg, #gayman, #gaymen, #gayboy; 69/250, 27.6%). Few Instagram posts contained only male-associated hashtags (eg, #men; 39/250, 15.6%) or only female-associated tags (eg, #girlboss, #girlpower; 7/250, 2.8%). Very few posts contained race- or ethnicity-related hashtags (11/250, 4.4%), such as #BLACKPOWER and #latinxpower. Even fewer posts contained transgender-associated tags (e.g., #trans, #transgirl, #translatina; 5/250, 2%). No posts contained tags related to heterosexuals or injection drug users.

*RQ3: How did the textual content of PrEP-related posts vary by source (eg, individual accounts versus organization accounts)?* Instagram posts from organizations were more likely to describe who can use PrEP, compared to those posted by individuals, with *X*^2^_1_=9.7 (N=248) and *P*=.002. Almost one-third (62/193, 32.1%) of the posts from organizations described who can use PrEP, compared with 11% (6/55) of the posts from individuals. Conversely, Instagram posts from individuals were more likely to mention the adverse effects of PrEP, compared to the posts by organizations, with *X*^2^_1_=24.8 (N=248) and *P*<.001. More than 20% (12/55, 21.8%) of the posts from individuals mentioned PrEP adverse effects, compared to 3% (6/193) of the posts from organizations.

Regarding source attribution differences, Instagram posts from organizations were more likely to cite information from the CDC or other federal sources, compared to those posted by individuals, with *X*^2^_1_=5.3 (N=248) and *P*=.02. More than 10% (24/193, 12.4%) of the posts from organizations cited the CDC, compared to 2% (1/55) of the posts from individuals. Conversely, Instagram posts from individuals were more likely to cite information from a PrEP manufacturer compared to the posts by organizations, with *X*^2^_1_=9.8 (N=248) and *P*=.002. Almost 20% (10/55, 18.2%) of the posts from individuals mentioned Gilead or Merck, or their representatives, compared to 5% (10/193) of the posts from organizations. No other statistical differences were found.

## Discussion

### Principal Results

This research study is among the first to review Instagram posts for textual content specifically related to PrEP. We found that PrEP awareness/information (eg, posts defining PrEP) comprised the largest content focus; gay men comprised the priority population most commonly represented in these posts; organizations and individuals differed somewhat in terms of their post contents. These findings are further contextualized below.

### Comparison With Prior Work

Our results, similar to those reported in studies involving PrEP-related content on Twitter [27] and on YouTube [23,24], suggest that a broad range of topics are mentioned in PrEP-related posts on Instagram. Our finding that awareness-related posts featuring PrEP definitions represent the largest content focus is consistent with previous research results regarding PrEP messages on Twitter. Schwartz and Grimm offered possible explanations for why this may be the most prominent form of PrEP content on social media [27] and identified potential reasons for low prescription rates [45] and promotion of PrEP [46], along with the uncertainty conveyed in online news stories about PrEP appearing in the United States [42].

Uncertainty about health-related topics can drive information seeking [47] and if clear PrEP-related information is unavailable through traditional information sources, the uncertainty surrounding PrEP may prompt some individuals to consult alternative sources (including social media) to fulfill their informational needs. This notion is supported by Breen et al [25] who found increases in Twitter posts focusing on PrEP (as well as other health-related topics) in recent years. Young adults (especially those aged 18-24 years) may turn to social media as most individuals in this age group are already daily users of platforms such as Instagram [20]. Owing to the sensitive nature of PrEP-related information, these young adults may seek this information and other sexual health–related information from online sources (including social media), rather than from health care providers, or family and friends [48]. This could be viewed positively as organizations seem to be utilizing Instagram to provide PrEP-related information; however, as described in the following paragraphs, PrEP-related posts from organizations differ significantly from those of individuals in ways that may influence their impact on PrEP uptake and adherence.

One of the interesting findings from this research is that the current population-related focus appears to be on gay men, as the MSM population is the most commonly hashtagged priority population in the posts (eg, #gayman, #gaymen, #gayboy), although these individuals are not necessarily bisexual men. As rates of HIV diagnoses remain high among gay men, PrEP promotion messaging should focus on this important priority population. However, given that the rate of insertive condomless anal sex acts with casual partners is statistically significantly higher in bisexual men compared to gay men [49], a PrEP promotion focus on bisexual men is sorely lacking in this social media space. Such a focus is important, as research indicates that focusing on HIV in cities with high numbers of bisexual men (men who have sex with men and women) may have the dual effect of improving the health of the bisexual community and the health of MSM populations that have a high burden of HIV [50].

We also found that very few Instagram posts contained race- or ethnicity-related co-occurring hashtags, and even fewer posts contained transgender-associated co-occurring tags. Given that Black individuals account for a higher proportion of new HIV diagnoses and people living with HIV, compared to other races/ethnicities, people of color represent important priority populations in terms of HIV risk and PrEP promotion [51]. This is especially true for men and women, as Black men represented 31% of the new HIV diagnoses in the United States, and Black women are approximately 16 times more likely to receive a diagnosis of HIV infection compared to White women [51,52]. In addition to a weak social media focus on PrEP for people of color, we found that only 2% (5/250) of the posts contained transgender-associated tags although transgender persons are at a very high risk for HIV infection [53]. Lastly, we found no PrEP promotion focusing on heterosexual adults or injection drug users in Instagram posts, both of which represent groups for which PrEP is indicated in the United States [8]. The lack of PrEP promotion on Instagram for bisexual men, people of color, transgender persons, heterosexual adults, and people who inject drugs remains a critical missed opportunity.

Our results also show some differences in the source characteristics of the PrEP-related posts as well as the textual content shared by organizations and individuals. Organizations comprised the vast majority (193/250, 77.2%) of PrEP-related Instagram posts, a stark difference compared to that reported by McLaughlin et al [26] who stated that organizations were less common creators of tweets focusing on PrEP. Organizations are significantly more likely to share information regarding who can use PrEP. This focus may coincide with the advocacy goals of HIV nonprofit organizations seeking to leverage social media as a platform for intervening and expanding organizational capacity to increase PrEP awareness, disseminating educational material, and enhancing engagement with members of the public [54,55]. If part of these public engagement and outreach efforts involves partnerships with federal agencies, it may help explain why organizations (compared to individuals) in our data are more likely to cite the CDC and other federal sources in their PrEP-related content. Another possibility is that these organizations are more likely to mention federal agencies as reliable sources for scientific research on PrEP in their content to offer clarity and reduce public uncertainty about PrEP [27,56,57]. In contrast, individuals were more likely to include PrEP-related content focusing on the adverse effects of PrEP and were also more likely to mention manufacturers such as Gilead or Merck, or their representatives. One explanation for this result may be that individuals are more likely to focus on the adverse effects of PrEP and cite PrEP manufacturers as part of larger public discussions surrounding consumer experiences with the pharmaceutical drug industry and drug safety. Prior research suggests that social media can be a rich source for these public discussions [58]. These findings highlight the perceived differences between posts made by PrEP users and those organizations that develop or promote PrEP. We recommend that organizations work with communities related to PrEP or sexual health influencers on Instagram to develop and maintain explicit partnerships for implementing communication strategies aimed at reducing barriers to PrEP uptake and adherence (eg, stigma, lack of knowledge about adverse effects, and costs). This strategy has been examined by previous research into Instagram suggesting that influencers provide several techniques for disseminating information that may be less possible for organizations (eg, word-of-mouth dissemination and celebrity status updates) [59-61].

### Limitations

This research should be considered within the context of its limitations. First, the Instagram posts included in this research are only those that are publicly available. Although this is consistent with the data showing that most social media users report setting all their social media accounts to private [62], it presented a limitation to our study, as we were unable to generalize our findings beyond publicly accessible posts. Second, although we followed a methodical process for identifying hashtags to use in our search for posts, we may have excluded some hashtags, resulting in missed posts pertaining to PrEP. Third, of the 193 posts coded as stemming from organizations, 49 originated from a single account (Prevention305), which represents 25% of that organizational subgroup. This may have generally skewed the findings for organizations toward Prevention305’s PrEP awareness campaign goals. Fourth, as this study was our first attempt to apply a predefined codebook used in textual analyses of Twitter data to Instagram, we have not reported on the data extracted from photos or videos. The only exception was when we described the basic topology of the images (ie, whether the image was a photo, an infographic, or a video). This necessitates a slightly different application of our methodology than what was stated as the purpose of this study and may be utilized in future projects to expand on the findings highlighted in the current paper. Lastly, because we focused our review on English-only posts, we may have missed important Instagram content related to Spanish-speaking people (eg, Latinx).

Despite these limitations, our study exhibits some strengths. This study is among the first to examine PrEP-related textual content on Instagram. Despite its popularity, especially among young adults, Instagram remains an understudied platform, and the current study begins to address this gap in the extant literature. In addition, although our sample only includes publicly available posts, to the extent that as these posts are indeed reflective of the public PrEP-related information environment on Instagram, they may point toward types of PrEP-related content that Instagram users seeking information about PrEP are likely to encounter. Future research may confirm this if interviews or surveys are conducted to determine the most common types of PrEP-related content Instagram users recall encountering during active searches for PrEP information. Public health professionals may also consider the publicly available user comments for PrEP-related content posted on Instagram to gauge user reactions. These user comments may offer insights into how priority populations may respond to PrEP-related content serving as the basis for future PrEP-related interventions. Future studies should also strive to examine such content across social media platforms, including Instagram, Facebook, and Twitter.

### Conclusions

The National AIDS Strategy’s call to more clearly articulate the science surrounding HIV risk and prevention is more fully addressed by first understanding the current public information environment surrounding PrEP. The present study seeks to begin answering this call by offering a snapshot of how PrEP is being discussed (and by whom) on one of the most popular social media platforms. In addition, this study responds to the National AIDS Strategy’s recommendation to develop campaign strategies that leverage the unique properties of emerging digital technologies by laying the foundation for big data approaches that may be applied to glean insights for messaging in PrEP campaigns and interventions. These findings highlight the additional efforts required to reach the National AIDS Strategy’s goal of improved communication surrounding PrEP. The small number of Instagram posts that feature PrEP highlight a less than optimal level of engagement, and the current study should serve as a call for investigators to utilize emerging tools such as Instagram more effectively to engage priority populations in conversations around PrEP.

## Abbreviations

CDC: Centers for Disease Control and Prevention
FTC: emtricitabine
GBM: gay and bisexual men
HPV: human papillomavirus
MSM: men who have sex with men
PrEP: pre-exposure prophylaxis
RQ: research question
SD: standard deviation
STDs: sexually transmitted diseases
TDF: tenofovir disoproxil fumarate
